# ANLN and UBE2T are prognostic biomarkers associated with immune regulation in breast cancer: a bioinformatics analysis

**DOI:** 10.1186/s12935-022-02611-0

**Published:** 2022-05-16

**Authors:** Yu Xiao, Zhiqin Deng, Yongshen Li, Baoting Wei, Xiaoqiang Chen, Zhe Zhao, Yingjie Xiu, Meifang Hu, Murad Alahdal, Zhenhan Deng, Daping Wang, Jianquan Liu, Wencui Li

**Affiliations:** 1 Department of Breast and Thyroid Surgery, Shenzhen Second People’s Hospital/The First Hospital Affiliated to Shenzhen University, Shenzhen, 518000 China; 2 Hand and Foot Surgery Department, Shenzhen Second People’s Hospital/The First Hospital Affiliated to Shenzhen University, 3002 Sungang West Road, Shenzhen, 518000 China; 3 Department of Sports Medicine, Shenzhen Second People’s Hospital/The First Affiliated Hospital of Shenzhen University Health Science Center, Shenzhen, 518000 Guangdong China; 4 Department of Pathology, Shenzhen Second People’s Hospital/The First Hospital Affiliated to Shenzhen University, Shenzhen, 518000 China; 5 Department of Biomedical Engineering, Southern University of Science and Technology, Shenzhen, 518055 China

**Keywords:** Breast cancer, Luminal type of breast cancer, Prognostic biomarkers, Immune regulation, T cells

## Abstract

**Objectives:**

To screen and verify differential genes affecting the prognosis of breast cancer.

**Methods:**

Breast cancer gene expression datasets were downloaded from the GEO database, and original data were analyzed in R. The TIMER database was used to analyze the relationship between ANLN and UBE2T and immune cell infiltration.

**Results:**

Ten hub-key genes were identified, and survival analysis showed that *UBE2T* and *ANLN* were upregulated in breast cancer and their upregulation was associated with a poor prognosis. *ANLN* and *UBE2T* upregulation was associated with the prevalence of Th1 and Th2 cells, shifting the Th1/Th2 balance to Th2 in Basal and Luminal-B breast cancers, which indicates a poor prognosis (P < 0.05).

**Conclusion:**

ANLN and UBE2T are potential biomarkers for predicting the prognosis of breast cancer.

## Introduction

Breast cancer is the most common cancer in women, with more than 2 million new cases each year [1–3]. Despite the availability and good results of several treatment options according to tumor molecular subtype, many patients have a poor prognosis [4, 5].

The prognosis of breast cancer is associated with tumor phenotype. A European study that followed-up patients for 10 years showed that the 10-year survival rate was 81.4% for luminal A, 77.3% for luminal B, 76.4% for HER2-positive, and 70.0% for triple-negative breast cancer (TNBC) [6]. A similar study assessed the 15-year relative survival rate of 205,827 women (in the same cancer registry) diagnosed with stage I–III breast cancer between 1989 and 2008. This study found that the survival rate was closely related to age and tumor stage [7]. Although relatively good prognoses have been observed, especially for luminal type breast cancers, many patients show endocrine therapy resistance and recurrence [8].

Gene mutation patterns and gene expression profiles in breast cancer have been extensively analyzed, and important pathways related to breast cancer carcinogenesis have been elucidated [9, 10]. Genes associated with breast cancer prognosis and the response to drugs have been identified [11, 12]. Studies show that tumor-infiltrating lymphocytes are associated with prognosis and mediate the treatment response to chemotherapy and immunotherapy in pan-cancers including breast cancer [13, 14]. However, because of differences in biological characteristics between different subtypes, the prognostic differences in breast cancer are not fully understood. To identify accurate biomarkers for predicting the prognosis of breast cancer, we screened differentially expressed genes (DEGs) in breast cancer using bioinformatics analysis and analyzed their functions and the correlation with immune cell infiltration, which may play a crucial role in the outcome of breast cancer patients.

## Materials and methods

### Data collection and analysis

Data downloads, statistical analyses, and visualization were performed using R (version 3.6.3). The GEOquery [15] and sva packages were used for data downloads and to remove inter-batch differences, respectively. Differential gene expression analysis was performed using the limma package. The umap package was used for UMAP analysis, the ggplot2 package was used for visualization, and the ComplexHeatmap package [16] was used for heat map visualization. The GSE43358, GSE65194, GSE50567 datasets were downloaded from GEO (https://www.ncbi.nlm.nih.gov/geo/) using the GEOquery package. These datasets contained 17 cases of normal breast tissue, 72 cases of TNBC (ER^−^/PgR^−^/HER2^−^), 53 cases of HER2 positive (HER2^+^) breast cancer, 45 cases of luminal A (ER^+^/HER2^−^/histological grade 1), and 40 cases of luminal B (ER^+^/HER2^−^/histological grade 3) breast cancer.

### Immune infiltration correlation

The correlation between *ANLN*, *UBE2T*, and immune infiltration in breast cancer was examined using The Cancer Genome Atlas (TCGA) database. The ssGSEA function in the R package “GSVA”[17] was used to calculate immune infiltration in breast cancer. The immune cells studied included activated dendritic cells (aDCs), B cells, CD8 cytotoxic T cells, DC eosinophils, immature DCs (iDCs), macrophages, mast cells, neutrophils, CD56 bright natural killer (NK) cells, CD56 dim NK cells, NK cells, plasmacytoid DCs (pDCs), T cells, T helper cells, T central memory (Tcm), T effector memory (Tem), T follicular helper (Tfh), T gamma delta (Tgd), Th1 cells, Th17 cells, Th2 cells, and T regulatory cells (Tregs).

### PPI network construction

The STRING (https://string-db.org/) online database (version 11.0) was used to construct a protein–protein interaction (PPI) network. Then, the MCODE plug-in component of Cytoscape software (version 3.7.2) was used to sort and filter the key modules.

### Immunohistochemistry

Human breast tumor specimens were obtained from the Breast and Thyroid Department, Shenzhen Second People’s Hospital, Guangdong Province. All experiments involving human tissues were performed in accordance with the principles of the Declaration of Helsinki and were approved by the Institutional Review Board of the Shenzhen Second People’s Hospital. The primary antibodies and antigen retrieval protocols used were as follows: ANLN: bs-7738R, primary antibody, rabbit polyclonal antibody against aniline; Bioss Antibodies (http://www.bioss.com.cn/). UBE2T: 10,105–2-AP, UBE2T/HSPC150 polyclonal antibody; Proteintech (https://www.ptglab.com/).

### Statistical analysis

The IOD/area ratio was calculated using ImagePro Plus 6.0. Statistical analyses were performed using GraphPad Prism 8.0.1 software. Statistical significance was evaluated using two-tailed t-tests and defined as *p < 0.05, **p < 0.01, and ***p < 0.001.

## Results

### Screening of differentially expressed genes in luminal breast cancer

**Fig. 1:**
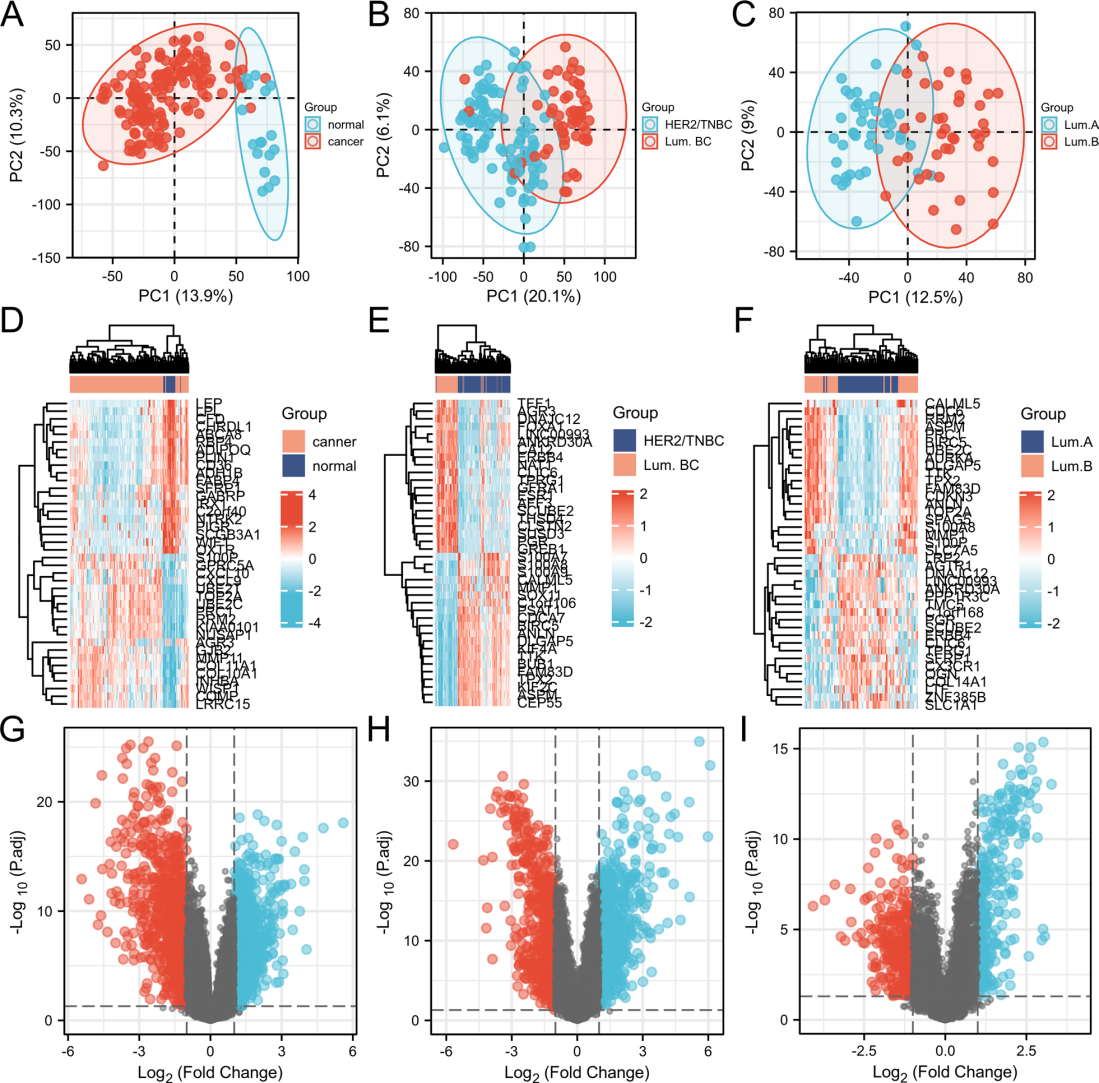
Screening of differential genes for luminal breast cancer. **A**–**C** Principal component analysis in three intersections of breast cancer groups. **D**–**F** Top 40 differentially expressed genes from different intersections are shown in heatmaps. **G**–**I** Differential genes from different intersections are shown in the volcano plot

Analysis of overlapping genes from breast cancer and luminal type breast cancer identified 40 DEGs. Principal component analysis (PCA) was used to analyze DEGs (Fig. 1A–C), and a heatmap according to different groups as well as a volcano plot were generated for the top 40 genes (Fig. 1D–I). Table 1 shows the signaling pathways corresponding to the 40 DEGs.

**Table1:** Signal pathways corresponding to the 40 differential genes

ID	Description	p. adjust
hsa04110	Cell cycle	9.334E − 38
hsa03030	DNA replication	9.3648E − 11
hsa04114	Oocyte meiosis	1.3613E − 10
hsa03440	Homologous recombination	4.7677E − 09
hsa03460	Fanconi anemia pathway	4.7677E − 09
hsa04218	Cellular senescence	1.3877E − 08
hsa04914	Progesterone-mediated oocyte maturation	2.656E − 08
hsa05166	Human T-cell leukemia virus 1 infection	7.5356E − 06
hsa03430	Mismatch repair	7.5356E − 06
hsa04115	p53 signaling pathway	9.9277E − 06

**Fig. 2:**
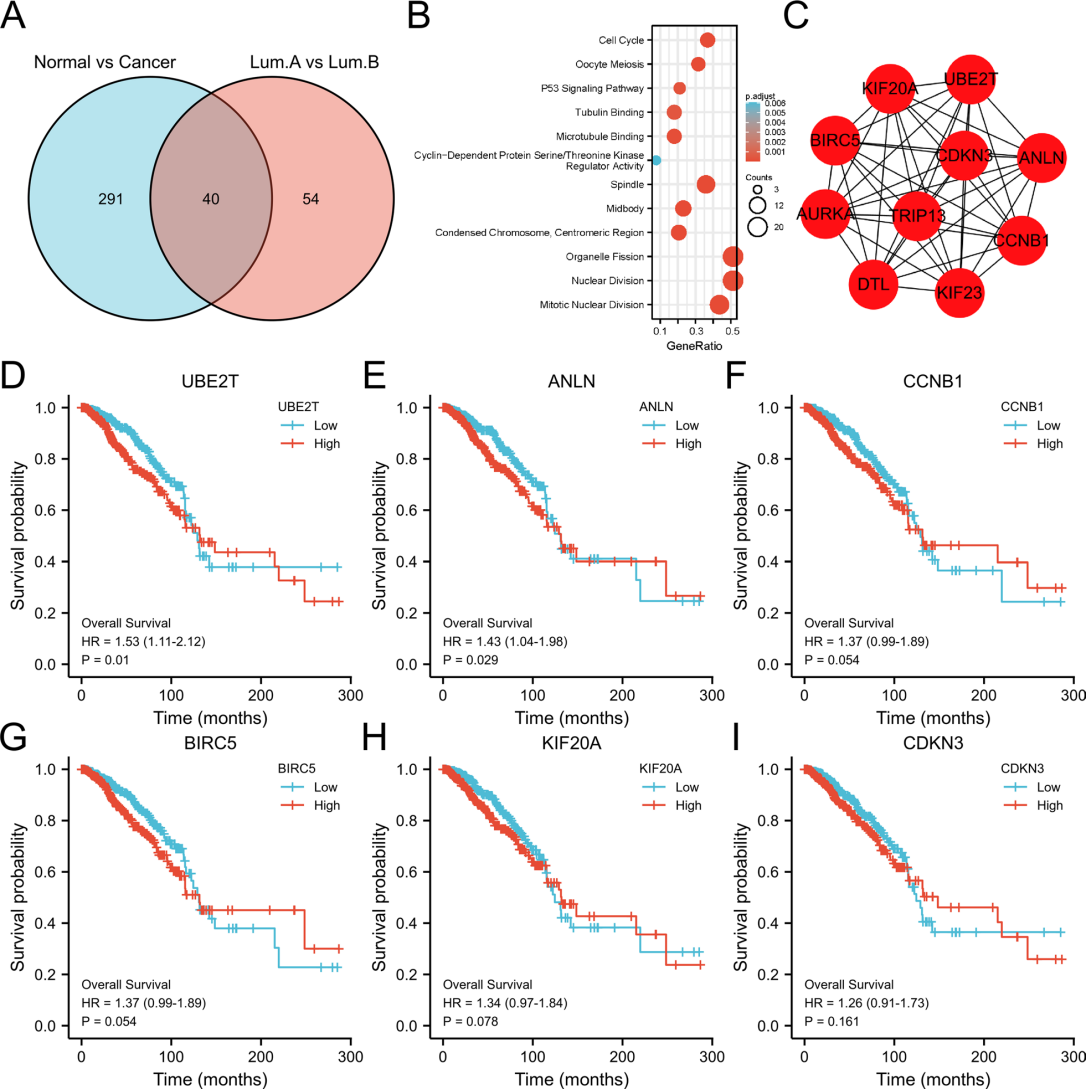
Association between *ANLN* and *UBE2T* expression and cancer prognosis. **A** The intersection of breast cancers and luminal type breast cancers identified 40 differentially expressed genes. **B** Enrichment of signaling pathways of 40 differentially expressed genes. **C** The Cytoscape MCODE module was used to screen 10 hub-key genes. **D**–**I** Kaplan–Meier analysis of overall survival in TCGA. Results with log-rank P < 0.05 are shown only for *ANLN* and *UBE2T*

The enrichment of signaling pathways for 40 DEGs identified 12 pathways (Fig. 2A, B), indicating a potential mechanism for these genes. The Cytoscape MCODE module was used to screen 10 hub-key genes (Fig. 2C). Survival analysis showed that only *UBE2T* and *ANLN* were significantly associated with survival (P < 0.05) (Fig. 2D, E).

### Pan-cancer *ANLN* and *UBE2T* expression analysis

**Fig. 3:**
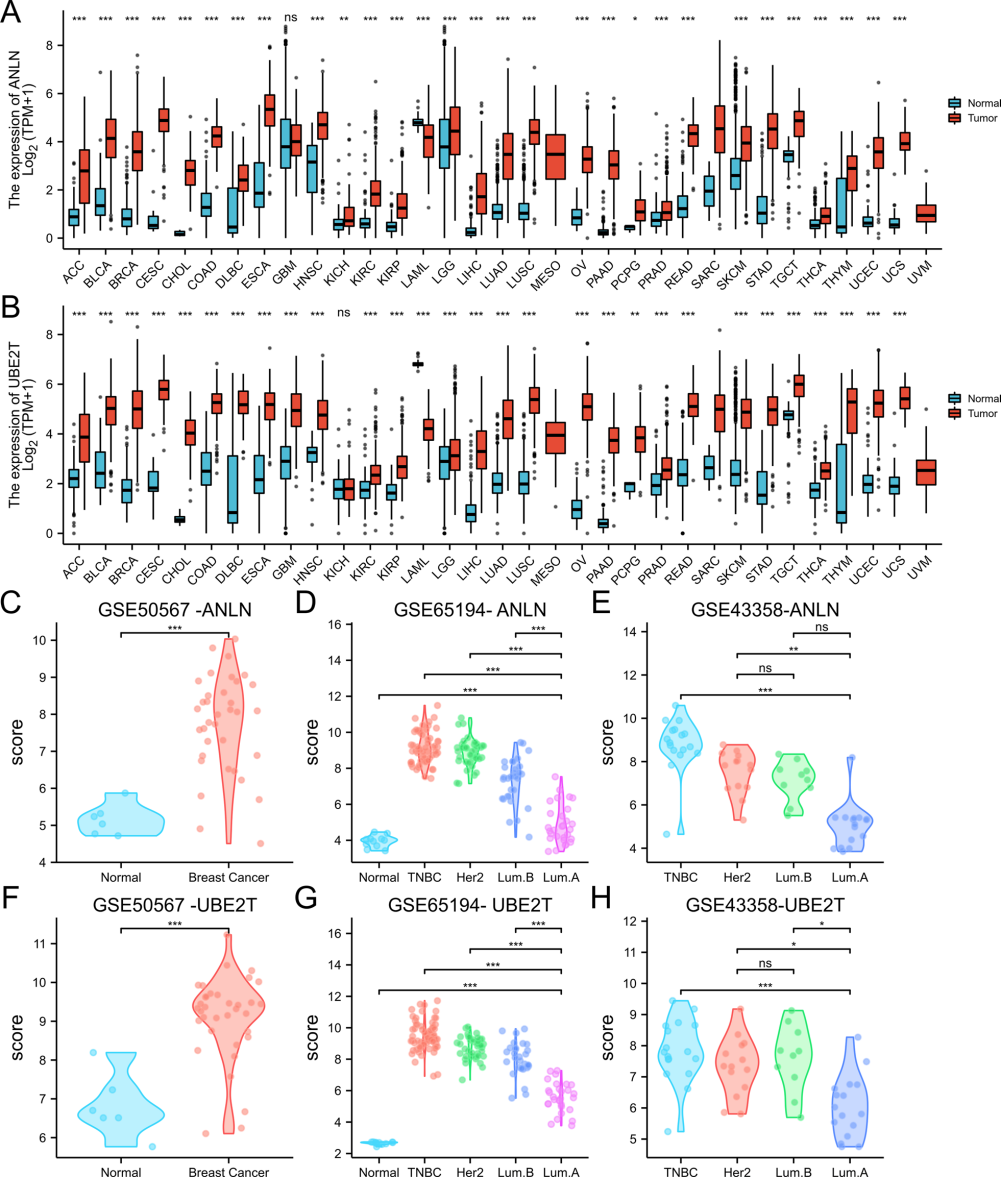
Pan-cancer *ANLN* and *UBE2T* expression analysis. **A** *ANLN* expression in tumor and normal tissues from TCGA pan-cancer data. **B** *UBE2T* expression in tumor and normal tissues from TCGA pan-cancer data. **C** *ANLN* expression in normal tissues and breast cancer tissues from GSE50567. **D**, **E** *ANLN* expression in different subtypes of breast cancer tissues from GSE65194 and GSE43358. **F** *UBE2T* expression in normal tissues and breast cancer tissues from GSE50567. **G**, **H** *UBE2T* expression in different subtypes of breast cancer tissues from GSE65194 and GSE43358

We assessed *ANLN* and *UBE2T* expression in 33 tumors from TCGA, including ACC, BLCA, BRCA, CESC, CHOL, COAD, DLBC, ESCA, GBM, HNSC, KICH, KIRC, KIRP, LAML, LGG, LIHC, LUAD, LUSC, MESO, OV, PAAD, PCPG, PRAD, READ, SARC, SKCM, STAD, TGCT, THCA, THYM, UCEC, UCS, and UVM (Fig. 3A, B). Analysis of the GSEA50567, GSEA65194, and GSEA43358 datasets showed that *ANLN* and *UBE2T* expression was higher in breast cancer than in normal tissues (P < 0.05) (Fig. 3C, D, F, G) and higher in TNBC and HER2 overexpressing subtypes than in luminal A and luminal B subtypes (P < 0.05) (Fig. 3E, H).

### Correlation of *ANLN* and *UBE2T* with immune cells and enrichment analysis

**Fig. 4:**
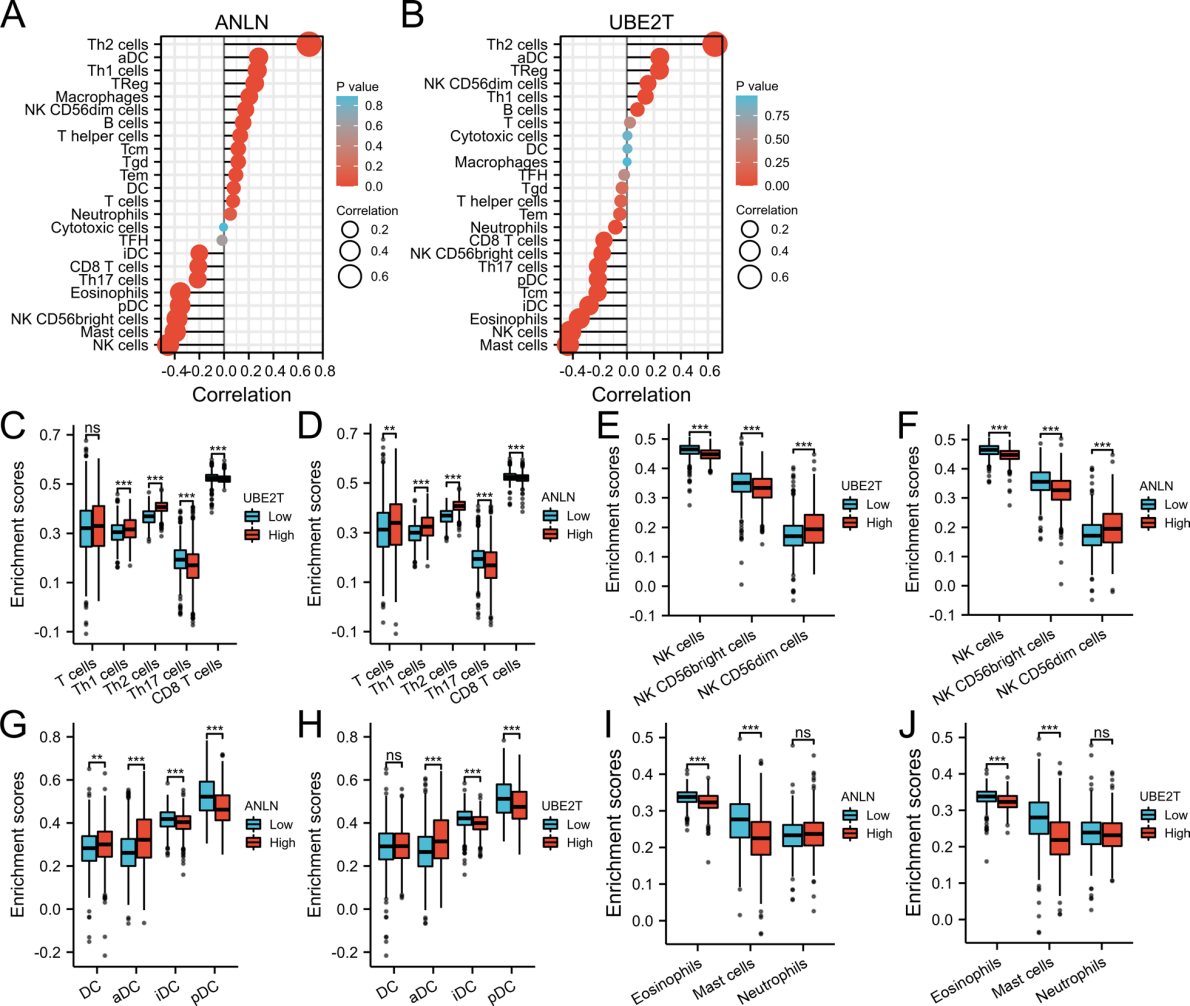
Correlation and enrichment analyses of *ANLN* and *UBE2T*. **A**, **B** The correlation between *ANLN*, *UBE2T*, and immune cells. **C**–**J** The correlation between the expression of *ANLN*, *UBE2T*, and the enrichment scores of different immune cells

The association of *ANLN* and *UBE2T* with immune cells was analyzed. Th2, aDC, Th1 and Tregs were positively correlated with *ANLN* and *UBE2T* expression, whereas NK and mast cells were negatively correlated (Fig. 4A, B). The enrichment scores indicated that T cells, Th1 cells, Th2 cells, CD56dim NK cells, DCs, and aDCs were enriched in association with high *ANLN* expression (Fig. 4D, F, G; P < 0.05). Th1 cells, Th2 cells, CD56dim NK cells, and aDCs were associated with high *UBE2T* expression (Fig. 4C, E, H; P < 0.05). By contrast, Th17 cells, CD8 T cells, NK cells, CD56bright NK cells, iDCs, pDCs, eosinophils, and mast cells were not enriched in association with high expression of *ANLN* and *UBE2T* (Fig. 4C–J; P < 0.05). These results indicate that *ANLN* and *UBE2T* are associated with immune-related pathways.

### Correlation between immune cell infiltration, *ANLN*, and *UBE2T*

**Fig. 5:**
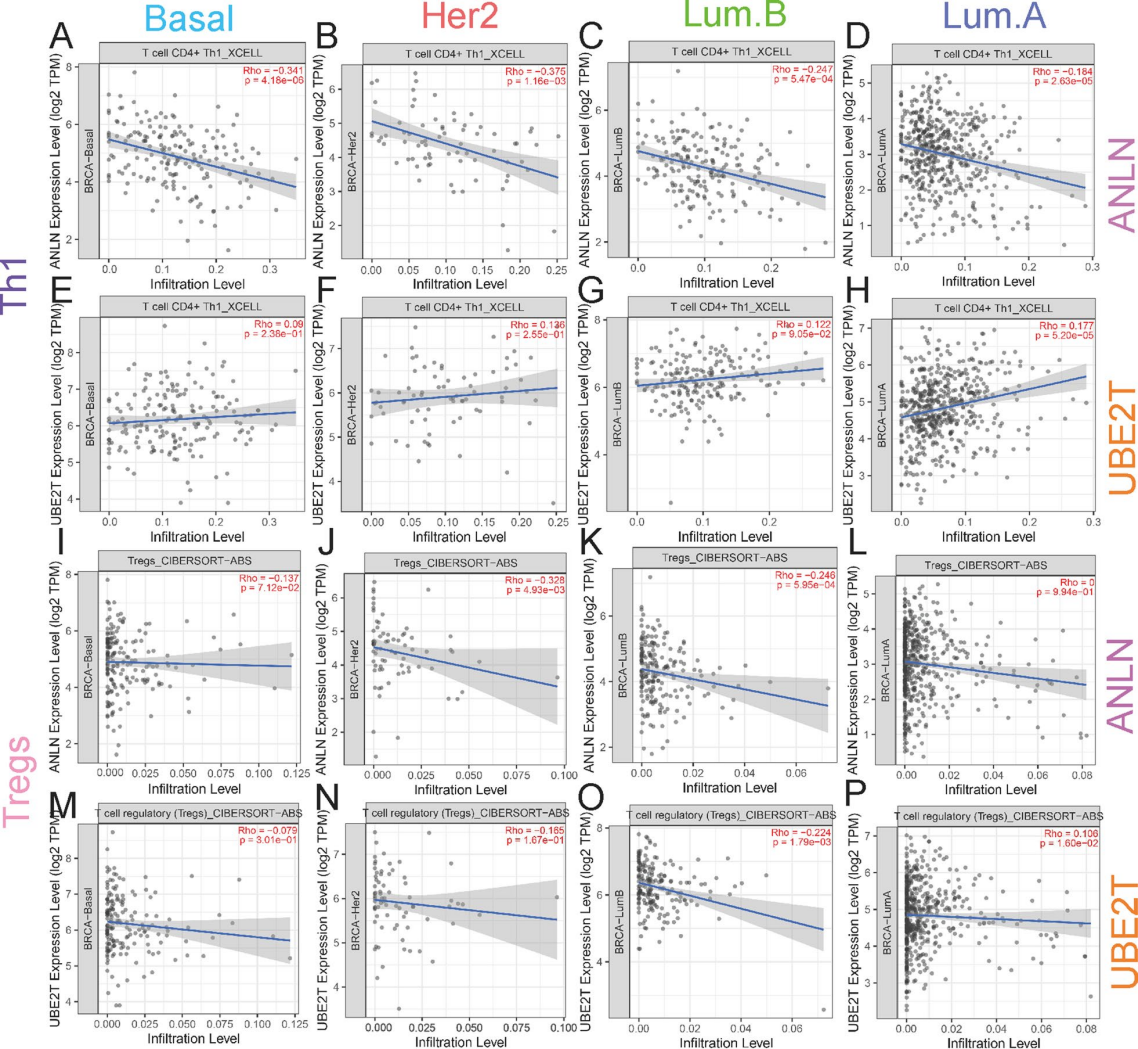
Correlation between *ANLN* and *UBE2T* expression and Th1 and Treg cells. **A**–**D** Correlation between *ANLN* expression in different breast cancer subtypes and Th1 infiltration levels using the XCELL algorithm. **E**–**H** Correlation between *UBE2T* expression in different breast cancer subtypes and Th1 infiltration levels using the XCELL algorithm. **I**–**L** Correlation between *ANLN* expression in different breast cancer subtypes and Treg infiltration levels using the CIBERSOFTABS algorithm. **M**–**P** Correlation between *UBE2T* expression in different breast cancer subtypes and Treg infiltration levels using the CIBERSOFTABS algorithm

**Fig. 6:**
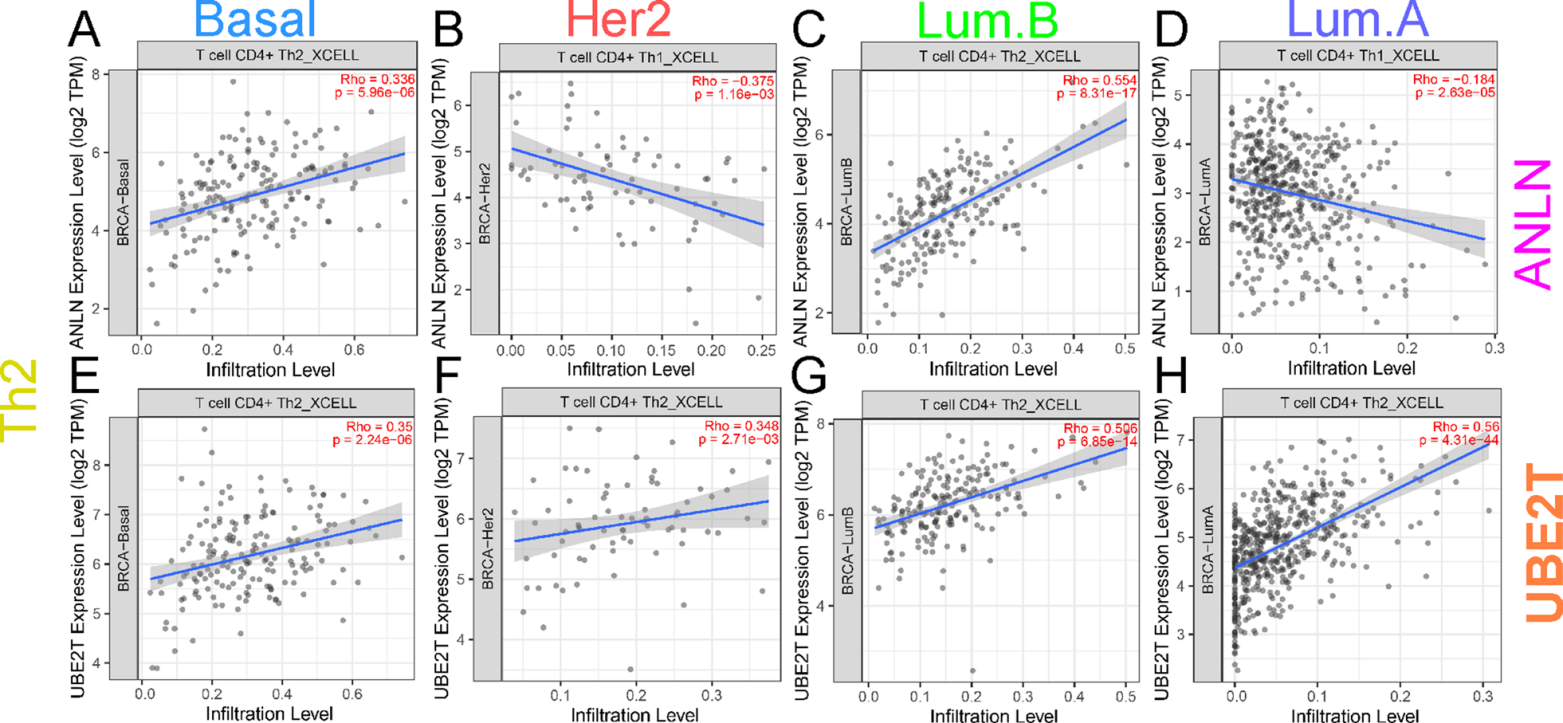
Correlation between *ANLN* and *UBE2T* expression and Th2 cells. **A**–**D** Correlation between *ANLN* expression in different breast cancer subtypes and Th2 infiltration levels using the XCELL algorithm. **E**–**H** Correlation between *UBE2T* expression in different breast cancer subtypes and Th2 infiltration levels using the XCELL algorithm

We further assessed the immune cell infiltration score using the TIMER2 (http://timer.cistrome.org/) database for different subtypes of breast cancer. The results showed that Th1 cell infiltration levels were low in the high-*ANLN* expression group in all subtypes (Fig. 5A–D). Th1 cell infiltration level was high in the high-*UBE2T* expression group only in the luminal A subtype (Fig. 5H). Tregs infiltration levels were low in the high-*ANLN* expression group and HER2 overexpression group in the luminal B subtype (Fig. 5J, K). Tregs infiltration levels were low in the high-*UBE2T* expression group only in the luminal B subtype (Fig. 5O). Th2 cell infiltration levels were high in the high-*ANLN* expression group in the basal-like and luminal B subtypes, and low in the high-*ANLN*, HER2 overexpression, and luminal A subtypes (Fig. 6A–D). Th2 cell infiltration levels were high in the high-*UBE2T* expression group in all subtypes (Fig. 6E–H). This indicates that the expression of *ANLN* and *UBE2T* affects immune cells in different subtypes of breast cancer. The Th1 and Th2 infiltration results were consistent in luminal B type breast cancer. Taken together, these findings indicate that the expression of *ANLN* and *UBE2T* is associated with immune cell infiltration and the immunosuppressive microenvironment in different subtypes of breast cancer.

### Validation of the expression of ANLN and UBE2T in breast cancer

**Fig. 7:**
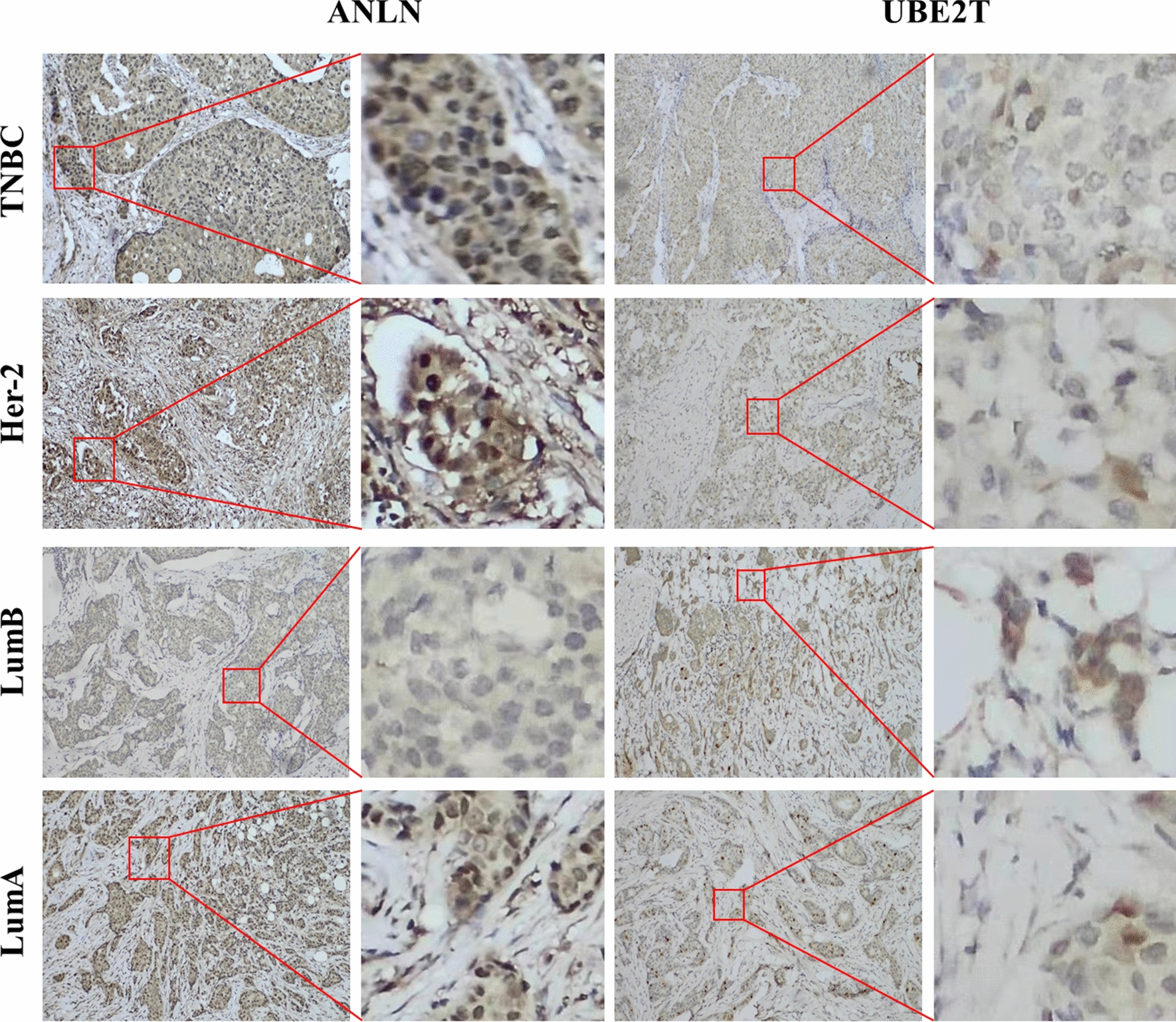
Expression of *ANLN* and *UBE2T* in breast cancer tissues. Representative immunohistochemistry images showing ANLN expression in breast cancer tissues (first and second column). Representative immunohistochemistry images showing UBE2T expression in breast cancer tissues (third and fourth column)

Paraffin samples were used to examine the expression of ANLN and UBE2T in breast cancer tissues by immunohistochemistry, and three samples of each subtype of breast cancer were included. The results confirmed that the expression of ANLN and UBE2T was detectable in all types of breast cancer (Fig. 7).

## Discussion

ANLN and UBE2T are widely expressed in various cancer tissues. ANLN is associated with the progression of lung, pancreatic, and cervical cancers [18–20], whereas UBE2T is associated with proliferation in hepatocellular carcinoma, glioblastoma, gastric cancer, and renal cell carcinoma [21–25]. In breast cancer, limited studies have shown that the expression of *ANLN* is associated with a poor prognosis and promotes doxorubicin resistance. Similarly, UBE2T promotes the proliferation, invasion, and glycolysis of breast cancer cells by regulating the PI3K/AKT signaling pathway [26–30]. However, the exact roles of these genes in breast cancer development remain unclear. Elucidating the role of ANLN and UBE2T in breast cancer is thus important.

In this study, we showed that ANLN and UBE2T are expressed in multiple cancerous tissues. Analysis of TCGA database confirmed the pan-cancer expression of these two genes. In addition, *ANLN* and *UBE2T* were highly expressed in breast cancer, especially in TNBC and HER2-positive breast cancers compared with luminal A and luminal B breast cancers. The expression of the ANLN and UBE2T proteins was confirmed by immunohistochemistry using paraffin-embedded specimens.

The expression of *ANLN* and *UBE2T* was associated with immune cell infiltration, which varies according to subtype. Tumors with immune cell infiltration are associated with different survival patterns based on estrogen receptor (ER) status. Tumors lacking immune cell infiltration have the worst prognosis in ER-negative samples, whereas they are associated with intermediate outcomes in ER-positive disease. Among the cell subpopulations studied, Tregs and M0 and M2 macrophages were strongly associated with a poor prognosis, but not with ER status. CD8 + T cells and activated memory T cells were associated with a favorable prognosis in ER-negative tumors. T follicular helper cells and memory B cells were associated with pathologic complete response and ER-negative neoadjuvant chemotherapy therapy, suggesting a role for humoral immunity in mediating the response to cytotoxic therapy. Unsupervised cluster analysis using immune cell proportions revealed eight tumor subgroups, defined primarily by the balance between M0, M1, and M2 macrophages, with distinct survival patterns based on ER status and associated with patient age at diagnosis [31]. In the present study, immune cell infiltration analyses showed that Th1 was negatively associated with *ANLN* in all types of breast cancer, whereas it was positively associated with *UBE2T* in luminal A breast cancer. Th2 was significantly positively associated with *ANLN* in TNBC and luminal B breast cancer and significantly positively associated with UBE2T in all subtypes of breast cancer. These results indicate that the upregulation of ANLN and UBE2T in different subtypes leads to different polarizations of the Th1/Th2 balance, and the polarization towards Th2 cells may result in disease progression. Animal model experiments suggest that shifting the Th1/Th2 balance toward Th1 results in the inhibition of breast cancer [32, 33]. Mulligan et al. confirmed that a high proportion of Th1 cells in breast cancer tissue is associated with a favorable outcome by immunohistochemistry [34]. Conversely, a Th1/Th2 balance shifted towards Th2 cells causes breast cancer progression. A large study that divided breast cancer samples into different clusters based on immunogenomics features showed that the cluster with the lowest Th1/Th2 ratio had the poorest outcome in the survival analysis [35]. However, different immune-related patterns may indicate different mechanisms underlying the function of *ANLN* and *UBE2T* in different breast cancer subtypes.

The regulatory mechanisms of ANLN and UBE2T in tumors were investigated previously. Cross-species genome comparison suggested that the genomic changes of UBE2T and UBE2C are related to the occurrence, development, and invasion of breast cancer. In addition, UBE2T is overexpressed in breast cancer tissues. Analysis of the C Bioportal database showed that UBE2T is amplified in approximately 12% of breast cancer tumors and is associated with adverse outcomes in basal-like and luminal breast cancer patients [36, 37]. Similarly, there are differences in the expression of ANLN in different subtypes of breast cancer. The key genes related to ANLN regulation also differ between different subtypes of breast cancer [38, 39]. Although our study explored the expression and potential roles of these two genes in breast cancer, their exact effect on cancer progression in different subtypes of breast cancer remains to be clarified.

Because this study used second-generation sequencing data of mRNA provided by TCGA database, the expression of ANLN and UBE2T was analyzed at the mRNA level, which does not completely represent the mRNA expression level results. However, this study included a large sample size as well as objective and complete clinical data with high credibility, which is representative to a certain extent and can provide clues and a basis for further research to explore the function and mechanism of ANLN and UBE2T in the development and progression of breast cancer subtypes.

## Conclusion

**Fig. 8:**
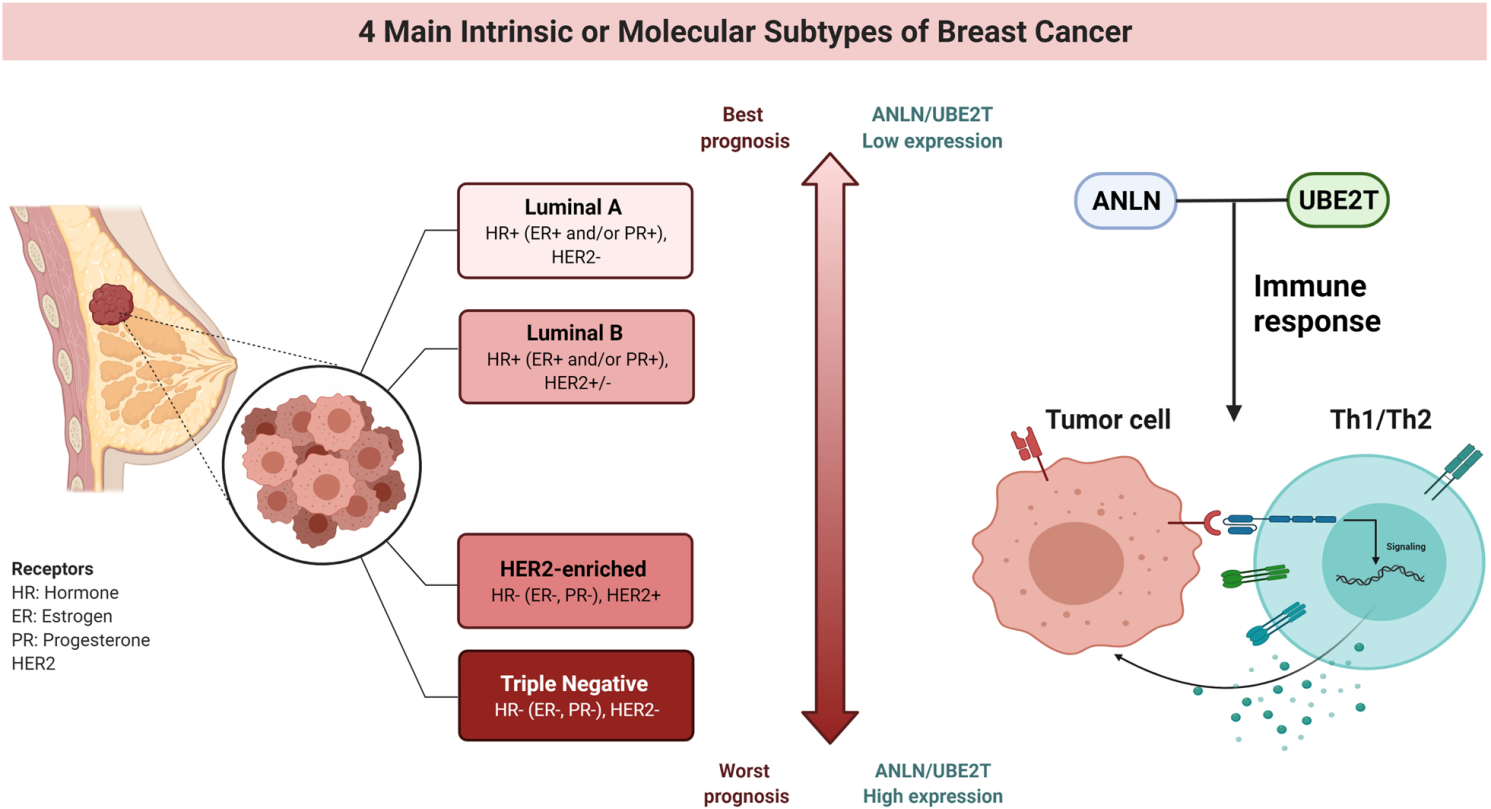
ANLN and UBE2T are potential prognostic biomarkers in breast cancer. *ANLN* and *UBE2T* were consistently associated with Th1 and Th2, shifting the Th1/Th2 balance to Th1 in Luminal-B breast cancer, which indicates a poor prognosis. Created with BioRender.com

Upregulation of the expression of ANLN and UBE2T is associated with a worse prognosis of breast cancer. ANLN and UBE2T are potential prognostic biomarkers and novel therapeutic targets in breast cancer. The interaction between UBE2T and Th1/Th2 may interfere with a positive breast cancer prognosis. These findings should be further investigated (Fig. 8).

## Future perspectives

Future studies should be performed with a greater number of samples to verify the correlation between the expression of ANLN and UBE2T in different subtypes of breast cancer and their association with prognosis.

Studies in cells or animals should be performed to elucidate the signaling pathways through which ANLN and UBE2T regulate the Th1/Th2 balance.

In addition, antibodies or drugs targeting ANLN and UBE2T may be developed to analyze their potential for the treatment of cancer.

## Summary points

An overlap of genes from breast cancer and luminal type breast cancer identified 40 differentially expressed genes.

The enrichment of signaling pathways for 40 differentially expressed genes showed that 12 pathways were involved, including those related to the cell cycle and nuclear division. Survival analysis showed that only the *UBE2T* and *ANLN* genes were significantly associated with a poor outcome. The expression of *UBE2T* and *ANLN* was higher in the TNBC and HER2-over-expression subtypes than in the luminal A and luminal B subtypes and may lead to a poor prognosis. The expression of ANLN and UBE2T influence immune cells in different subtypes of breast cancer. The expression of ANLN and UBE2T in all types of breast cancer was confirmed by immunohistochemistry.

## Data Availability

Gene expression profiles, clinical information and mutation data of THCA in this study are available from the public database (TCGA, https://portal.gdc.cancer.gov/) and GEO (https://www.ncbi.nlm.nih.gov/geo/).
